# *‘Choice should be made through… educated decisions not regressive dictates’*: discursive framings of a proposed ‘sugar tax’ in Bermuda: analysis of submissions to a government consultation

**DOI:** 10.1186/s12992-022-00877-7

**Published:** 2022-10-21

**Authors:** Sarah Williams, Sarah E. Hill, Oyinlola Oyebode

**Affiliations:** 1 Warwick Medical School, University of Warwick, CV4 7AL Coventry, UK; 2 SPECTRUM research consortium (Shaping Public Health Policies to Reduce Inequalities and Harm), University of Edinburgh, EH8 9AG Edinburgh, UK; 3 Office for Health Improvement and Disparities, London, UK; 4 Sydney School of Public Health, University of Sydney, Sydney, Australia

**Keywords:** Commercial determinants of health, Sugar tax, Taxation, Food policy, Global health governance, Small island states

## Abstract

**Background:**

Several governments have introduced taxes on products with high sugar content as part of their obesity prevention strategies. Bermuda is the first jurisdiction to apply such measures in the Caribbean – a region of small island developing states and territories with high obesity prevalence and substantial reliance on imported food products. This study examines how commercial and health actors framed the proposed introduction of a 75% import tariff on high-sugar products, based on written submissions to the Bermudan government.

**Methods:**

Eleven submissions containing written comments were analysed with reference to their framing of the proposed import tariff, the ‘problem’ of obesity, and the relationship between the two (including alternative policy approaches for tackling obesity).

**Results:**

Key emergent frames were *complexity*, *partnership*, *products*, *personal responsibility*, *affordability* and *evidence*. Respondents favoured different framings, depending on whether they supported or opposed the proposed import duty. Commercial actors were universally opposed, presenting obesity as a ‘complex’ problem that would be better addressed through government-industry partnerships (a framing particularly favoured by international and regional business associations). Increased product range and an emphasis on personal responsibility were also positioned as policy alternatives. Health actors expressed partial support for the proposed sugar tax, although this was tempered by a perceived lack of evidence where the proposal differed from sugar taxes introduced elsewhere. Like commercial respondents, health actors framed obesity as a ‘complex’ problem and emphasised the need for other measures, including efforts to address the affordability of fruits and vegetables.

**Conclusion:**

In responding to a proposed ‘sugar tax’ in Bermuda, commercial actors opposed the proposal and stated a clear preference for ‘partnership’ approaches to tackling obesity. Commercial responses were dominated by local businesses (with only two responses received from international or regional business associations), perhaps reflecting Bermuda’s reliance on tourism and hospitality and the specificity of the proposed intervention (that is, an import tariff rather than an excise tax). The much smaller number of responses from health actors suggests limited civil society capacity. Nevertheless, the Bermudan government successfully introduced a 75% tariff on high-sugar imports, demonstrating the potential for policy innovation to address obesity in small-island jurisdictions.

**Supplementary Information:**

The online version contains supplementary material available at 10.1186/s12992-022-00877-7.

## Background

Obesity contributes to a range of non-communicable diseases (NCDs), which collectively account for more than 70% of deaths worldwide.[1] Excess consumption of sugar is linked with obesity and other diet-related NCDs,[2] prompting calls to protect population health by reducing sugar intake.[3] Fiscal measures such as taxes on products with high sugar content are known to be effective in lowering consumption.[4–6] Such ‘sugar taxes’ – most of which are specific to sugar-sweetened beverages (SSBs) - have now been introduced in many regions including the Americas, Europe, sub-Saharan Africa, Southeast Asia and the Pacific.[7].

The appropriateness of sugar taxes has been a focus of debate in jurisdictions considering their introduction, including in Latin America,[8] the USA,[9] Australia,[10] the UK [11] and Ireland.[12] This controversy is perhaps unsurprising, given SSBs and other ultra-processed foods constitute a massive transnational business [13] - meaning producers have a vested interest in preventing or delaying effective measures to reduce consumption.[14] A comparative study of Mexico, Chile and Colombia found the influence of such corporations posed a significant barrier to the introduction of taxes on SSBs, particularly where there was a lack of transparency in establishing the policy agenda.[8] In contrast, taxes on SSBs were more likely to be adopted where they had wide support across government, civil society and community organisations.

The role of corporate actors in NCDs has come under increasing scrutiny with reference to the ‘commercial determinants of health’, defined by Kickbusch et al. as *“strategies and approaches used by the private sector to promote products and choices that are detrimental to health”*.[15] These strategies – best understood for the tobacco industry - include a range of discursive tactics intended to discourage policy action by exaggerating the costs and diminishing the benefits of proposed legislative changes.[16] Multinational companies producing SSBs and other ultra-processed food products have been shown to use such strategies in an effort to obstruct public health measures.[17, 18] Arguments against regulation may be advanced by the corporations themselves, but are also commonly represented by business associations in the context of both local and international policy discussions.[8, 19] Increased corporate permeation, which may facilitate such discursive tactics (as well as comprising a political strategy in itself), is associated with reduced implementation of NCD policies.[20].

The link between obesity and ultra-processed food consumption is particularly marked in the Caribbean region, where most populations are highly dependent on imported foods (half the countries in this region are thought to import more than 80% of all food consumed).[21] In Bermuda - a small island territory at the edge of the Caribbean – over a third of all adults are thought to be obese.[22] While data on overall sugar intake are lacking, a third of adults report drinking at least one or two sugary drinks each day, with much higher intake in some groups (e.g. a daily intake of three to four sugary drinks is reported by a third of those with low incomes and over half of Black Bermudans).[22] At the same time, fewer than one in five Bermudans eat five servings of fruit and vegetables per day (almost half report just one to two servings per day).[22].

In 2018, Bermuda’s government proposed to increase tariffs on imported sugar and sugary products; in other words, to introduce a form of ‘sugar tax’.[23] This would take the form of an import duty of at least 75% on all high-sugar products - including soft drinks, confectionary and pure sugar. While the duty would be paid by importers in the first instance, the government anticipated the cost would be passed on to consumers in the form of higher product prices. As part of the consultation, the government asked respondents to comment on whether the import duty should be set at 75% or higher, with 150% suggested as an alternative.[23] It is also worth noting that Bermuda’s ‘sugar tax’ is not limited to sugar-sweetened beverages (the more common approach globally) but applies to any food products with high sugar content, similar to Hungary’s ‘junk food’ tax on any foods high in sugar and/or salt. [24]

This project aims to examine how commercial and health actors sought to influence the policy debate around Bermuda’s proposed ‘sugar tax’, based on analysis of substantive written submissions. This adds to existing literature addressing the discursive strategies used by different actors in relation to food policy [8, 10, 12, 19] while providing a novel contribution in its focus on a small-island context where the majority of food is imported and on a tax applying to all high-sugar products (rather than specifically to SSBs).

## Methods

This study analysed written responses to Bermuda’s 2018 ‘Sugar Tax Consultation’.[25] The Bermudan government received 351 responses to this consultation, including 35 submitted on behalf of organisations and 316 submitted by individuals (conversation with D.S. Kendell, January 2021). Most of these responses were very brief, comprising short answers to specific questions presented via the consultation’s online portal; however, 11 respondents provided more substantive written comments comprising a page or more of free text (email from health@gov.bm, January 2021). The analysis presented here is based on these 11 written submissions as providing a suitable dataset to examine respondents’ framing of the proposed sugar tax and the ‘problem’ it purports to address. Seven of the 11 submissions came from commercial organisations, including one international business association (the International Council of Beverages Associations), one regional business association, and five local businesses. Two submissions came from local health organisations; and two were written by self-declared health professionals living and working in Bermuda (Table 1, for further details see supplementary table).

Qualitative analysis of submissions used a form of frame analysis – that is, we focused on how respondents presented or ‘packaged’ relevant problems (such as obesity) and proposed solutions (such as the sugar tax) in conceptual terms.[26, 27] In focusing on the framing of policy debates, this approach is less concerned with the evidence underpinning different policy options than with the values, assumptions and discursive strategies used by actors seeking to influence policy discussions.[26] As Niederdeppe and colleagues explain, “[a]dvocates and opponents of specific policies seek to shape policy debates by framing the policy issue in ways they see as favourable to their positions…”.[9] An understanding of which frames or arguments are common to particular actors can thus offer insights into the political economy of health policy and its context-specific dynamics, and has been widely used by researchers examining debates around sugar taxes and other proposed interventions in the food system.[8–10, 12, 19].

Submissions were read and coded in a two-stage process. In the first stage, the content of the submissions was organised into a range of preliminary themes via a process of open coding including both deductive and inductive elements. This process was informed by an *a priori* understanding of the consultation document, which included 13 specific questions that respondents were invited to answer; therefore some themes mirrored the topics of these questions, while additional themes emerged from the submissions themselves. This first-stage was undertaken using the principles of thematic analysis [28] in which the first author coded data according to common themes arising from the data. This was done alongside regular discussions with co-authors to clarify the coherence and relevance of emergent themes across the submissions. In the second stage of coding, preliminary themes were reviewed and organised into broader frames according to how respondents were presenting the ‘problem’ of poor diet and obesity and potential policy ‘solutions’ to this problem. This was informed by knowledge of common frames reported in the wider literature, some of which were evident in our findings. This process was undertaken by the first author in consultation with co-authors. All data were coded in Nvivo 12.

**Table 1:** Source of substantive written submissions to Bermuda’s proposed sugar tax

Categories of respondents	Number of responses
Commercial actors	
- *International business associations*	*1*
- *Regional business associations*	*1*
- *Local businesses*	*5*
Health actors	
- *Local health organisations*	*2*
- *Local health professionals (responding as individuals)*	*2*
Total	11

## Results

Analysis of the 11 written submissions identified six broad frames used by commercial and health actors in discussing policy responses to obesity (Table 2). While the first of these frames (*complexity*) was used by both commercial and health respondents, the remaining five frames were specific to either commercial actors (who opposed the proposed sugar tax) or health actors (who generally supported the sugar tax, although with some reservations). Our findings are therefore organised according to broad categories of respondent (commercial and health), in order to highlight the relationship between actors’ policy preferences and the frames used to support these positions.

**Table 2:** Key frames and arguments evident in submissions on Bermuda’s proposed sugar tax

Frame	Related arguments	Respondents using this frame
Complexity	Obesity is a complex problem driven by multiple factors There is no single intervention that will ‘fix’ obesity	Commercial actors (international, regional and local), health actors
Partnership	Commercial actors have already taken steps to reduce obesity, thus demonstrating their commitment as responsible social actors A sugar tax risks undermining existing partnerships between business and government, particularly in relation to Bermuda’s tourism industry	Commercial actors (international, regional and local)
Products	Ultra-processed high-sugar products also deliver health benefits (providing energy and nutrients, boosting wellbeing) These products are not inherently ‘unhealthy’; rather, health problems arise when individuals fail to moderate their consumption	Commercial actors (international, regional and local)
Personal responsibility	Policy responses should focus on enabling informed individual choice	Commercial actors (international, regional and local)
Affordability	Policies to tackle obesity should include measures to increase the affordability of healthy foods	Health actors (local)
Evidence	We should focus on policy measures for which there’s existing evidence of effectiveness	Health actors (local)

## Commercial actors

### Complexity

Responses from commercial actors acknowledged the necessity of acting to address obesity, but universally opposed the sugar tax as a means of doing so. In general, obesity was framed by these actors as a complex, multifaceted problem:

*The causes of obesity however are numerous and are a complex combination of too much food consumption and far too little physical exercise by modern society. (Local business)*

One possible outcome of this framing is an implicit rejection of obesity as an ‘industrial epidemic’,[29] driven by the activities of the ultra-processed food industry. Instead, causation is attributed to multiple complex factors:

*Obesity is largely the result of an imbalance in excess energy consumption and too little energy expenditure over time… obesity has been fuelled by a variety of complex environmental, social, economic, behavioural, and/or other factors. (International business association)*

This framing may thus serve to both deflect attention from commercial activity as a driver of obesity and diminish the potential effectiveness of an intervention focusing on just one element of this complex causation. For commercial actors, the ‘complexity’ framing thus supported their opposition to the proposed sugar tax on the basis this was not addressing most of the (complex) drivers of obesity.

Alongside this ‘complexity’ framing (used here to cast doubt on the efficacy of sugar taxes as a response to obesity), commercial actors employed three framings that supported their suggestions for alternative policy responses. These focused on **partnership** approaches between the health sector and industry, the role of the market in providing a range of **products** (emphasising the health benefits of those products), and the importance of **personal responsibility** in addressing the causes of obesity.

### Partnership

This frame suggests that obesity will be solved through a partnership approach. Commercial actors strongly favoured this frame – i.e., for business to be present alongside government in developing responses to obesity – with public-private collaboration depicted as the most promising way forward. This argument was reinforced by the complexity framing: the rationale being that, since obesity is caused by a complex system, it requires a ‘holistic’ approach and the involvement of industry to develop suitably innovative ‘solutions’. The partnership framing was evident in submissions across the range of commercial respondents, with the scale of such partnerships ranging from international (“the global community”) to local (”all Bermudians”):

*… members have long made robust commitments to help address some of the complex health and nutrition-related challenges facing the global community… These types of partnership-based initiatives are more effective than taxes in lowering the amount of calories consumed and making a positive impact on public health/obesity. (International business association)*

*Adopting a multi-stakeholder approach, including Industry-Government partnership is really important in achieving a team effort to address this important issue. The food industry has a key responsibility and members recommend to partner with the Government in addressing this issue with a holistic approach. (Regional business association)*

*… we must all work together to try to do everything possible to ensure a healthy future for all Bermudians (Local business)*.

The partnership framing presented the ultra-processed food industry as a responsible social actor whose voluntary actions demonstrated a collective commitment to reducing obesity:

*There is also a strong industry commitment regarding responsible communication to consumers, for example controlled marketing to children; innovation as well as renovation to significantly reduce levels of sugar, fat, saturated fat and salt; nutrition labelling to support consumers in making healthy choices; guidance on recommended serving sizes; and voluntary front of pack nutrition labelling. (Regional business association)*

In addition to positioning themselves as important partners in policy making, the International Council of Beverages Associations argued their industry delivered important economic benefits that also supported health. Taxation was thus presented as inappropriate since it would not only undermine partnership between industry and government, but would have a detrimental impact on health:

*The economic impact of selective taxation may also have detrimental effects on health. The economic growth created by beverage and retail industries contributes positively toward health outcomes… By singling out sugar and/or SSBs for discriminatory tax treatment, governments… are pursuing policies that have a disproportionate detrimental impact on the very populations they are supposed to help, and therefore may worsen health outcomes. (International business association)*

Local businesses placed particular emphasis on the threat the proposed sugar tax posed to existing partnerships. They argued the tax would damage tourism and hospitality, which were central to the Bermudian economy. By emphasising this centrality, local businesses effectively argued that a sugar tax would threaten the sustainability and even the identity of the Bermudan community:

*Bermuda must avoid any change that could hurt the hospitality and tourism industry as both are so imperative to our economy. (Local business)*

*At this point we really need to ask ourselves, “Are we truly in the hospitality industry or not?” If we accept the tax, I would suggest our answer was a loud “No!” and we better start looking for another pillar to our economy. (Local business)*

### Products

Commercial respondents also sought to present the market as a source of alternative ‘solutions’ to obesity and other health challenges. Arguments here emphasised the value of a diverse market providing options to address a range of needs, including products that were nutritious, pleasurable and beneficial sources of energy. Within this range a consumer would be able to select a varied and ‘balanced diet’.

Regional and local business actors described products high in sugar as an important source of energy, which in turn could promote exercise:

*Sugar sweetened beverages and other food products with added sugar contribute energy to consumers. (Regional business association)*

*Sports drinks, while containing added sugar, are formulated to help replenish the nutrients athletes lose (including sugars)… Sports drinks are marketed towards active consumers and encourage people to exercise, which is important to help improve the health of Bermudians. (Local business)*

Several respondents also sought to present these products as providing important nutritional value:

*Some of these products are fortified with other micronutrients (vitamins and minerals) that contribute to meeting these daily nutrient reference values. (Regional business association)*

*Putting a tax on “plain” sugars… will discourage the home made process of creating foods of potentially the best nutritional value of all. (Local business)*

*100% juices and milk-based beverages are inherently nutrient dense (International business association)*.

A related argument sought to present high-sugar products as beneficial for emotional wellbeing. As with the claims above, this argument suggests the proposed sugar tax may have unintended adverse health effects because consumption of these products can bring health benefits. It also implies that the products themselves are not unhealthy; rather, health risks (such as obesity) arise when people fail to consume them in moderation:

*The whole concept of the tax is to discourage people from partaking in a pleasurable experience which, in moderation, is not harmful… this is paternalistic governance at its most invasive (Local business)*.

*As a mother I actively reduce my children’s intake of sodas and ‘junk foods’, as an individual I’ve developed a healthy diet and increased my exercise programmes, as an employer I have implemented 90-day health and fitness programmes for my bakery team. All of these health initiatives allow for the moderate consumption of luxury foods. (Local business)*

Commercial respondents also queried the link between high-sugar products and obesity. Relevant arguments challenged the focus on sugar and questioned the likely efficacy of sugar taxes in managing obesity:

*… the intake of sugar has been decreasing in several countries - including Bermuda - yet the prevalence of overweight and obesity have either stayed the same or increased. (International business association)*

*… soda and sugary drink purchase and consumption has been in decline for several years in Bermuda (Local business)*.

### Personal responsibility

In highlighting alternative approaches to tackle obesity, commercial actors favoured interventions that ‘supported’ individuals to make informed choices and consume high-sugar products in moderation. This framing emphasises the role of personal responsibility in tackling obesity, thus diverting attention from high-sugar products and the businesses that produce and sell them. Proposed interventions included public education campaigns to inform dietary choices and promote physical activity, enabling people to exercise ‘choice’ and make ‘educated decisions’ about their diets:

*Choice should be made through free will guided by educated decisions not regressive dictates. (Local business)*

*Nutrition education combined with guidance on consumption levels is important so that consumers can understand how they can manage their intakes whilst enjoying a varied and balanced diet. (Regional business association)*

*To effectively combat obesity, ICBA thus believes public health efforts must focus more broadly on public education regarding the total diet, the importance of physical activity, and the provision of clear and fact-based nutrition information to consumers. (International business association)*

Responses from some local businesses appeared to question the government’s commitment to tackling obesity, suggesting they would invest more in education if this was a genuine policy goal:

*Perhaps if government initiated an awareness campaign… then the Government’s stated end goal of reducing staggering health care costs becomes more genuine to me. (Local business)*

## Health actors

Four responses from local health actors positioned obesity as a problem that needed tackling and at least partially supported the proposed sugar tax. As with commercial actors, health actors regarded the causes of obesity as complex and multifaceted. Unlike commercial respondents, however, there was notable variation in health respondents’ arguments, which generally did not position the causes of obesity in terms of distinct ‘problems’ or propose specific policy ‘solutions’. Respondents argued for ‘comprehensive’ policy responses that included measures to address the affordability of healthy foods. While the proposed sugar tax was seen as part of this solution, there was hesitancy towards setting higher or broader taxes than those had been tried elsewhere.

### Affordability

All health actors agreed that fiscal measures were an important policy lever in tackling obesity and supported a tax on sugar sweetened beverages. Such taxes were seen as supporting behaviour change:

*Other countries have found that tax on SSB’s [sic] has resulted in a change in behaviour in the consumption of these beverages. (Local health organisation)*

While broadly supportive of the proposed sugar tax, health actors’ enthusiasm was tempered by the sense that other measures were also needed. In particular, these actors favoured interventions to improve the affordability of healthy food options, particularly fruits and vegetables:

*Sugar tax is not going to solve the obesity and diabetes problem on its own. But it can be an important part of the solution. It’s about making the healthy choice the more affordable choice. (Local health organisation)*

*Measures to change the public’s behaviours will be most effective when the sugar tax is implemented in combination with multicomponent interventions… use of subsidies on fruit and vegetables could be an effective strategy to improve dietary quality and reduce the risk of negative substitution effects. (Local health professional)*

### Evidence

While health respondents universally agreed with the sugar tax in principle, all four queried specific aspects of the proposed tax – particularly where it varied from taxes introduced in other jurisdictions. In other words, there was a sense of hesitancy in relation to aspects of the tax that might be seen as innovative or going beyond existing policy precedents. Respondents often expressed caution or apprehension in relation to such innovation, which was framed as taking policy beyond the existing evidence base.

A particular focus of such hesitancy was the level of the proposed sugar tax. The consultation document asked whether a 75% duty rate was adequate, or whether a duty rate of 150% should be considered.[23] Some health respondents questioned whether 75% was too high, and all four argued that 150% would be excessive or even ‘draconian’ *–* despite acknowledging that higher taxes were more likely to results in behaviour change:

*Higher duty rates will make consumers more responsive to price change; however there is lack of clarity as to [the] selection process for the proposed 75% and 150% duty rates. (Local health professional)*

Health respondents also took varying positions over whether the proposed tax should be extended beyond sugar sweetened beverages to other foods with high sugar levels, an approach that is less common globally. One local health organisation (with a broad health mandate) was in favour of this approach, on the basis that a broad range of products contributed to the problem of obesity:

*…to impact obesity and chronic disease associated with habitual poor diets, both beverage and foods are a logical avenue. (Local health organisation)*

Other health respondents – including an organisation with a more specific disease focus - argued the tax should only be applied to sugar sweetened beverages. This position was supported with reference to what had been done – and found effective - in other jurisdictions (where taxes are specific to SSBs). Respondents who held this view made reference to the importance of evidence, and seemed hesitant about introducing measures for which there was not an established evidence base:

*Tax on other items has not been widely implemented in other countries and its effectiveness has not been determined in promoting positive health outcomes. (Local health organisation)*

## Discussion

Analysis of written submissions on Bermuda’s proposed sugar tax found respondents generally employed different framings, depending on whether they supported or opposed the tax. Responses from commercial actors tended to downplay the likely effectiveness of a sugar tax on the basis this addresses only one of the many ‘complex’ factors contributing to obesity. At the same time, commercial actors emphasised alternative policy approaches focused on frames of partnership between government and industry, product value, and an emphasis on personal responsibility. There were fewer responses from health actors, and – while these generally supported the proposed tax – this support was somewhat tempered. Like commercial respondents, health actors positioned the causes of obesity as ‘complex’ and indicated other policy measures were needed, although the most popular preferred alternative was also a fiscal measure (i.e. subsidisation of fresh fruits and vegetables). While health respondents were in favour of a sugar tax, there was a general reluctance to go beyond the available ‘evidence’ based on parameters of taxes shown to be effective in other jurisdictions.

In terms of articulating a clear policy position, submissions from business organisations consistently framed the ‘problem’ of obesity as one of poor personal choices, which corresponds with their preferred market-based ‘solutions’. In general, we found similar framings to those used by commercial actors elsewhere – including obesity as a ‘complex’ problem,[10, 19] ‘nanny state’, or government overreach [10] the importance of ‘personal responsibility’,[10] and a strong preference for ‘partnership’ between industry and government in developing policy responses to obesity.[19] Concerns over the negative economic impacts of the sugar tax were perhaps less evident here than in other analysis,[8–10] although they were present in submissions from local businesses which were particularly concerned about the potential impact of a sugar tax on Bermuda’s tourism and hospitality industries.

In contrast with sugar tax discussions in Ireland, [12] we found multiple examples of commercial actors employing the ‘personal responsibility’ frame - including in submissions from regional and international business associations. Campbell et al. suggest industry actors may be moving away from this frame as “the general public and government policy makers become more literate in the frames of obesity… [and] certain logics […] become widely suspect”.(p4) [12] This seems not to be the case in Bermuda, where the significance of hospitality and tourism may buttress against weakening of the personal agency argument. Economic dependence on tourism – a common issue in small-island jurisdictions – is commonly presented as an argument against regulation of unhealthy commodities in these contexts;[30, 31] and other researchers have also noted a reliance on ‘individual responsibility’ frames (including – at times – among policymakers) in discussing NCD prevention in the Caribbean.[31] As Campbell and colleagues suggest,[12] policymakers may find such arguments less convincing once they are aware of their strategic use by industry advocates [16] and the lack of evidence for the negative impacts of consumption taxes [32] and other public health measures [33–35] on tourism.

Health actors’ support for the sugar tax points to a more structural understanding of the drivers of obesity in Bermuda.[36] At the same time, their submissions were less clear in framing the sugar tax as a suitable policy response to a specific ‘problem’ (e.g. the wide availability of cheap, ultra-processed foods that are high in sugar and low in nutritional value). Several health respondents demonstrated impressive awareness of the application of sugar taxes in other jurisdictions, but their submissions did not convey unambiguous endorsement of the tax proposed by the Bermudan government. While agreeing with a sugar tax in principle, respondents’ support was contingent on Bermuda’s approach remaining within the bounds of what had been tried in other jurisdictions. Enthusiasm was also tempered by a sense that the sugar tax would not be sufficient in isolation from other measures to tackle obesity. This contrasts with a recent analysis of sugar tax discussions in Australia, where health advocates exhibited greater understanding of the effectiveness of taxes in reducing population consumption and less equivocal support.[10].

Efforts to influence policy discussions may be more effective where advocates articulate a clear match between the perceived problem and their preferred policy response.[27] Drawing on Kingdon’s theory of policy ‘windows’,[37] Katikireddi et al. examined advocates’ efforts to promote a minimum unit price for alcohol in Scotland and concluded their effectiveness at least partly rested on the successful presentation of the intervention as an appropriate ‘solution’ to a corresponding ‘problem’ (in this case, the ready availability of cheap alcohol).[38] In generating support for a sugar tax, it may be expedient for advocates to explicitly frame the relevant ‘problem’ in terms of the relative cheapness of ultra-processed foods.

Previous research has shown the ultra-processed food industry employs a range of tactics to resist regulatory controls on their markets,[39] with taxes on high-sugar products attracting the most robust opposition.[19] While industry submissions to Bermuda’s sugar tax consultation were limited in number, they did include responses from a regional and an international business association – suggesting broader interest in Bermuda’s approach, and potentially some concern that Bermuda’s example might encourage other jurisdictions to introduce similar taxes. The leadership of Mexico and other Latin American countries [7] suggests policy innovation may occur in those countries for whom obesity is a particular public health concern rather than those with the strongest track-records in regulating unhealthy commodity industries. This is likely to be of concern to the ultra-processed food industry given its increasing reliance on markets in emerging economies.[13].

Following the consultation, the Government of Bermuda subsequently introduced legislation imposing a 75% import duty on all high-sugar imports with the exception of fruit juices and milk products.[40] The legislation came into full effect in April 2019,[41] making Bermuda one of the first jurisdictions in the world (and the first in the Caribbean) to apply a tax to pure sugar and high-sugar foods as well as sugar-sweetened beverages.[42] The Bermudan government has committed to direct additional revenue from the sugar tax revenue into health promotion and disease prevention activities,[40] and has moved to eliminate import duties on some fruits and vegetables.[40] These represent important policy innovations in the context of emerging evidence on the effectiveness of sugar taxes for improving population diet,[7] warranting further study and evaluation. This is of particular importance as a recent analysis of NCD policy implementation found Small Island Developing States to be one of the geopolitical blocs most in need of support.[20] Early evaluation suggests that the policy has not proved popular, although has perhaps resulted in fewer purchases of taxed products. [43, 44]

Our research was limited to 11 substantive written submissions, which are unlikely to represent the views of all relevant stakeholders. In particular, the Bermudan government did not receive any submissions from transnational companies producing sugar-sweetened beverages and other high-sugar ultra-processed foods. This is perhaps surprising, given the potential for Bermuda’s import tariff on high-sugar products to be replicated in other jurisdictions. While coding and analysis of the data was primarily conducted by a single researcher, this process was supported via regular conversations with co-authors who also reviewed the emerging codes and sections of written responses. Identification of discursive frames is an interpretive process, meaning other researchers might have identified different frames from the responses analysed. Nevertheless, we believe this study makes a valuable contribution to existing literature examining discursive strategies in relation to a proposed ‘sugar tax’, particularly in its focus on a distinctive small-island context.

## Conclusion

Analysis of written submissions on a proposed ‘sugar tax’ in Bermuda found actors employed a range of discursive frames in order to support or oppose the tax. Commercial actors universally opposed the tax, positioning obesity as a ‘complex’ problem that would be more effectively addressed via a partnership approach in which the Bermudan government looked to business to innovate in providing health-enhancing products while focusing state efforts on educating consumers to make more responsible dietary choices. Such framings are consistent with the commercial sectors’ preference for minimising market regulation and deflecting attention from their role in driving NCD epidemics. Relatively few health actors made written submissions, perhaps reflecting somewhat limited civil society capacity in the context of a small-island jurisdiction. Those that did respond were broadly supportive of the sugar tax but were cautious about aspects that exceeded existing interventions in other countries. Our findings are broadly consistent with discursive analysis of sugar tax discussions in other policy settings, although we found ideas of ‘personal responsibility’ continue to prevail in a context where tourism and hospitality are particularly significant to the local economy and where local businesses may be more visible than transnational producers of ultra-processed food and beverages. Our findings have implications for health advocates seeking to maximise support for the use of fiscal measures such as sugar taxes in promoting health and preventing NCDs. These include the need to justify any policy innovation; prepare clear counter-arguments to industry framings on complexity and personal responsibility; and evaluate the impact of similar taxes on the tourism industry.

## Electronic supplementary material

Below is the link to the electronic supplementary material.

Supplementary Material 1

## Data Availability

The consultation responses analysed in the study are available from the Bermuda Department of Health (health@gov.bm).

## Abbreviations

NCD: Non communicable disease.
SSB: Sugar sweetened beverages.
