# Supplementary material for: ‘Choice should be made through… educated decisions not regressive dictates’: discursive framings of a proposed ‘sugar tax’ in Bermuda: analysis of submissions to a government consultation
Source: Global Health. 2022 Oct 21;18:89. doi: 10.1186/s12992-022-00877-7 (PMC9587584; doi:10.1186/s12992-022-00877-7)
Supplement: Supplementary file 1 — Supplementary Material 1 [file 12992_2022_877_MOESM1_ESM.docx]

Supplementary Table – Overview of respondents providing substantive written submissions to Bermuda’s 2018 consultation on a proposed sugar tax

| Respondent category | Respondent name | Description |
| --- | --- | --- |
| Commercial (international) | International Council of Beverages Associations (ICBA) | Members include Red Bull, Coca-Cola and several national and regional associations |
| Commercial (regional) | Trinidad and Tobago Manufacturer’s Association (TTMA) | Members listed in its food and beverage directory include Nestle, Angostura Ltd and British American Tobacco |
| Commercial (local) | Dunkley’s Dairy | Business in Bermuda supplying a range of food and drinks lines including local distribution of international brands |
|  | Gosling’s | Business in Bermuda manufacturing and retailing alcoholic and non-alcoholic drinks |
|  | Sweet Saak Bakery | Business in Bermuda retailing baked goods |
|  | Treats | Business in Bermuda retailing confectionary |
|  | R. M. Roberts | Wholesale candy business in Bermuda |
| Health (local organisations) | Bermuda Diabetes Association | Local health organisation |
|  | Bermuda Health Council | Regulatory health organisation in Bermuda |
| Health (local health professionals) | Dr Julia Darzi | Lecturer in nutrition and dietetics. Responding in an individual capacity |
|  | Shakira Warner | Population health specialist. Responding in an individual capacity |
